# Population Genetics of *Plasmodium vivax* in Four Rural Communities in Central Vietnam

**DOI:** 10.1371/journal.pntd.0004434

**Published:** 2016-02-12

**Authors:** Nguyen Van Hong, Christopher Delgado-Ratto, Pham Vinh Thanh, Peter Van den Eede, Pieter Guetens, Nguyen Thi Huong Binh, Bui Quang Phuc, Tran Thanh Duong, Jean Pierre Van Geertruyden, Umberto D’Alessandro, Annette Erhart, Anna Rosanas-Urgell

**Affiliations:** 1 National Institute of Malariology, Parasitology and Entomology (NIMPE), Hanoi, Vietnam; 2 Institute of Tropical Medicine (ITM), Antwerp, Belgium; 3 International Health Unit, Faculty of Medicine, University of Antwerp, Belgium; 4 Medical Research Council Unit (MRC Unit), Fajara, The Gambia; University of California San Diego School of Medicine, UNITED STATES

## Abstract

**Background:**

The burden of malaria in Vietnam has drastically reduced, prompting the National Malaria Control Program to officially engage in elimination efforts. *Plasmodium vivax* is becoming increasingly prevalent, remaining a major problem in the country's central and southern provinces. A better understanding of *P*. *vivax* genetic diversity and structure of local parasite populations will provide baseline data for the evaluation and improvement of current efforts for control and elimination. The aim of this study was to examine the population genetics and structure of *P*. *vivax* isolates from four communities in Tra Leng commune, Nam Tra My district in Quang Nam, Central Vietnam.

**Methodology/Principal Findings:**

*P*. *vivax* mono infections collected from 234 individuals between April 2009 and December 2010 were successfully analyzed using a panel of 14 microsatellite markers. Isolates displayed moderate genetic diversity (*He* = 0.68), with no significant differences between study communities. Polyclonal infections were frequent (71.4%) with a mean multiplicity of infection of 1.91 isolates/person. Low but significant genetic differentiation (*F*_*ST*_ value from -0.05 to 0.18) was observed between the community across the river and the other communities. Strong linkage disequilibrium ($I_{A}^{S}$ = 0.113, *p* < 0.001) was detected across all communities, suggesting gene flow within and among them. Using multiple approaches, 101 haplotypes were grouped into two genetic clusters, while 60.4% of haplotypes were admixed.

**Conclusions/Significance:**

In this area of Central Vietnam, where malaria transmission has decreased significantly over the past decade, there was moderate genetic diversity and high occurrence of polyclonal infections. Local human populations have frequent social and economic interactions that facilitate gene flow and inbreeding among parasite populations, while decreasing population structure. Findings provide important information on parasites populations circulating in the study area and are relevant to current malaria elimination efforts.

## Author Summary

In Vietnam, *Plasmodiu*m *vivax (P*. *vivax)* is the second most frequent human malaria parasite and a major obstacle to countrywide malaria elimination. Knowing the local parasite structure is useful for elimination efforts. Therefore, we analyzed, with a panel of 14 microsatellite markers, 234 *P*. *vivax* mono infections in blood samples collected from 4 communities in central Vietnam. Genetic diversity in the population was moderate; a high occurrence of polyclonal infections and significant linkage disequilibrium were detected, suggesting inbreeding or recombination between highly related haplotypes. In addition, both genetic differentiation and population structure was low and only detected between communities at each side of the river. Those results suggest gene flow between study communities with the river defining a moderate geographical barrier. Future studies should determine how this genetic variation is maintained in an area of extremely low transmission.

## Introduction

Vietnam has been extremely successful in decreasing the country’s malaria burden, thanks to the large scale implementation of control interventions such as insecticide-treated bed nets, indoor residual spraying, and prompt, free-of-charge diagnosis and treatment; the number of cases fell from 130,000 in 2004 to 27,868 in 2014 [1]. Malaria has been virtually eliminated from Northern and Southern Vietnam [1, 2]. In 2014, 80% of malaria cases occurred in nine “hot provinces” where annual incidence peaked at 3.1 cases per 1000, indicating a highly heterogeneous transmission, with hot spots of transmission (mostly in mountainous and forested areas) surrounded by areas of low transmission [1–5]. Vietnam aims at eliminating malaria by 2030 [6]. Such ambitious goal is threatened by *P*. *vivax*, whose characteristics (dormant liver forms that relapse weeks or months after clearance of the primary infection and gametocytes production before the occurrence of symptoms) together with the relative high occurrence of sub-patent and asymptomatic infections that remain undetected and thus untreated [3,7–10], make its transmission much more difficult to interrupt than that of *P*. *falciparum*. In addition, as already reported, elimination efforts are threatened by the emergence of drug resistance, for *P*. *falciparum* to artemisinin derivatives and partner drugs and for *P*. *vivax* to chloroquine (CQ) [2, 11, 12].

Current efforts to eliminate malaria are targeted to districts and communes reporting an increased number of malaria cases over time [2, 6]. In this context, understanding parasite genetic diversity and its population structure is relevant for (i) monitoring temporal changes in transmission following control efforts, (ii) elucidating the spatial distribution of parasite populations and predicting outbreaks, population resilience, and the spread of drug-resistant parasites, and (iii) identifying ecological and behavioral risk factors that can inform malaria control and elimination efforts [13–15].

A previous study conducted in Binh Thuan province in central Vietnam reported high levels of genetic diversity (average expected heterozygosity (*He)* = 0.86) and all infections being multi-clonal despite low transmission [13] (similar to what has been previously reported in South-East Asia) [16].

The aim of this study was to provide baseline data on the *P*. *vivax* parasite populations in four rural communities in the Vietnamese Quang Nam province.

## Materials and Methods

### Study site

**Fig 1:**
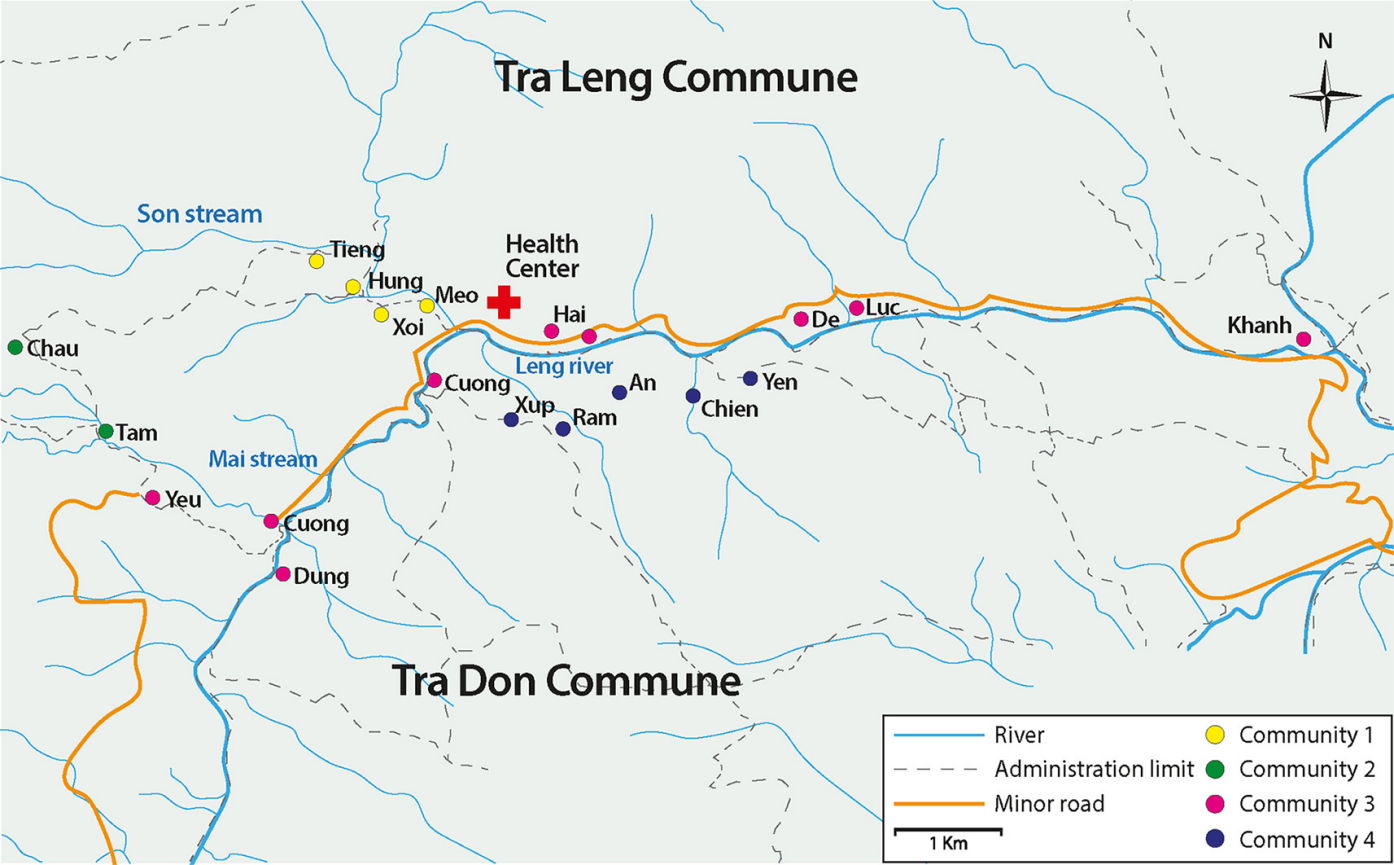
Study area in Nam Tra My district, Quang Nam, Vietnam.

Samples were collected from April 2009 to December 2010 in four communities (Fig 1) in the South Tra My district of Quang Nam, Central Vietnam during a prospective cohort study aiming to assess the short- and long-term efficacy of CQ and high-dose piperaquine (PQ) for the treatment of *P*. *vivax* mono-infections [12]. Detailed sociodemographic characteristics of the local population have been already reported elsewhere [3]. In 2009, the prevalence of malaria by light microscopy was 7.8%, while by polymerase chain reaction (PCR) prevalence was estimated at 22.6% (ranging from 16.4 to 42.5%), with a high proportion of *P*. *vivax* mono infections (43%). Sub-patent infections accounted for 58.7% of all infections, evidencing the existence of a substantial hidden human reservoir of malaria [3].

Malaria transmission is seasonal, and peaks during the rainy season (May to November). Based on data from the Provincial Malaria Station, between 2009 and 2013 the mean prevalence of *P*. *falciparum*, *P*. *vivax*, and mixed malaria cases for all age groups in the study area was 64.1%, 31.5%, and 4.4%, respectively [17–18]. The main malaria vectors in the area are *Anopheles dirus sensu stricto* and *An*. *minimus*, though *An*. *vagus*, *An*. *aconitus*, *and An*. *philippinensis* are also present [17–18].

### Sample collection

A finger prick blood sample was collected at day 0 (before treatment) for diagnosis by light microscopy and two blood spots were collected on grade 3 filter paper (Whatman Ltd., Springfield Mill, Maidstone, United Kingdom) for molecular diagnosis and microsatellite (MS) genotyping.

### Ethics statement

The study was approved by the National Institute of Malariology, Parasitology and Entomology in Hanoi, the Ministry of Health of Vietnam, and the review boards of the Institute of Tropical Medicine and Antwerp University Hospital (UZA) in Antwerp, Belgium. Adult participants (in case of minors one of the parents/guardians) provided written informed consent.

### Light microscopy diagnosis

Thick and thin film blood slides were stained with a 3% Giemsa solution for 45 minutes, and the number of asexual parasites was calculated following World Health Organization (WHO) guidelines [19]. Parasite density was estimated by dividing the number of asexual parasites for 200 white blood cells (WBCs) counted and expressed as the number of asexual parasites per microliter of blood, assuming 8000 WBC/μL. All blood slides were double-read by two technicians and in case of disagreement, slides were read by a third senior technician. The final results were expressed as the mean of the two closest results. A slide was declared negative if no parasites were found after counting 1,000 WBCs.

### Genotyping

DNA was extracted from filter paper blood spots cut into 5-mm-diameter disks with the QIAamp DNA Micro Kit following the manufacturer’s recommendations (Qiagen, Hilden Germany). *P*. *vivax* mono infections were confirmed by species-specific multiplexed semi-nested PCR, as described by Rubio et al. [20]. All samples were genotyped with 14 MS (MS1-MS10, MS12, MS15, MS20, and PvSal1814) following previously described PCR protocols [13–14]. The PCR products of four MS were pooled and analyzed by capillary electrophoresis in a 3730 XL ABI sequencer (Applied Biosystems) and 1200 Liz was used as the internal size standard. Negative samples were repeated once.

Allele calling was performed using GeneMarker version 2.4.0. After pooling capillary electrophoresis fsa files from all samples, a standard cut-off value of 500 relative fluorescence units was defined and peaks below this limit were considered background noise. In addition, all samples were double-checked manually to confirm true alleles. At each locus the predominant allele (the one giving the highest peak) and minor alleles within at least two-thirds of the height of the predominant allele were scored [13, 15, 21]. Only predominant alleles were used to define haplotypes to ensure an unbiased estimate of minor allele frequency in polyclonal infections [15, 22–24].

### Data analysis

Samples were defined as polyclonal if at least one locus presented more than one allele [13–14]. Polyclonal/locus (%) describes the percentage of samples identified as polyclonal by a given MS out of the total number of samples [13]. Multiplicity of infection (MOI), defined as the minimum number of different clones observed in a sample, was estimated by taking the maximum number of alleles at the two most polymorphic markers [13, 25]. Average MOI was defined as the sum of MOIs detected across all samples divided by the total number of samples. Average MOI and the proportion of monoclonal and polyclonal infections were compared with the Kruskal-Wallis and Pearson χ^2^ test, respectively. A value of *p* < 0.05 was considered significant. The predominant alleles in each sample were used to calculate the number of haplotypes by GenAlEx 6.5 [26]. Haplotypes that appear only once in the population were defined as unique haplotypes.

Genetic diversity, defined as the probability of observing different genotypes at a given locus in two unrelated parasites, was assessed by calculating the expected heterozygosity (*He*) for each community using the formula: He = [n/(n-1)*(1-∑p_*i*_^*2*^*]*, where *n* is the total number of alleles and *p* is the allele frequency. *He* ranges between 0 and 1, with values close to 1 indicating high genetic diversity [27]. Allelic richness, defined as the number of alleles per locus independently of sample size, was calculated using FSTAT v2.9.3 [28]. *He* and allelic richness were compared between communities using the Kruskal-Wallis test. The presence of bias due to false assignment of predominant haplotypes was investigated by comparing *He* in the database containing all the alleles and the database containing the predominant alleles [29].

A standardized index of association ($I_{A}^{s}$), calculated with LIAN v3.5, was used to assess the presence of multilocus linkage disequilibrium (LD) in the parasite population [30]. The significance of the $I_{A}^{s}$ estimate was assessed with Monte Carlo simulation using 10,000 random permutations of the data. To differentiate between clonal propagation and epidemic expansion, we compared LD in the predominant allele dataset and the unique haplotype dataset [31]. Pairwise LD was used to evaluate the physical linkage between loci located within the same contig using the G statistic in FSTAT v2.3.9 [28, 32].

Genetic differentiation between pairs of communities was estimated using a pairwise unbiased estimator of F-statistics FSTAT v2.3.9 with no assumption of Hardy-Weinberg equilibrium within samples [28, 32]. A matrix of *p* values corresponding to each pairwise *F*_*ST*_ was calculated after Bonferroni correction, with a value of *p* < 0.05 considered significant. Crude *F*_*ST*_ values were adjusted for sample size using Recode Data v. 0.1 [33] and standardized *F*_*ST*_ estimates were obtained by dividing crude *F*_*ST*_ values by adjusted *F*_*ST*_ values. *F*_*ST*_ estimates ranged from 0 (no genetic differentiation between communities) to 1 (full differentiation).

As a complementary approach, population structure was investigated using the software programs STRUCTURE v2.3.2 [34], CLUMPP [35], DISTRUCT [36], and GENODIVE [37]. STRUCTURE was used to identify clusters of genetically related samples. The number of clusters (*K*) was set from 1 to 10 with 10 replications per *K*, and 150,000 Markov Chain Monte Carlo steps after a burn-in period of 50,000 iterations using the admixture model. The loc-prior model was used for accurate inference of population and individual ancestry. Next, we used STRUCTURE HARVESTER v0.6.94 [38] to calculate the most likely number of *K* clusters. Additional data parsing and formatting of the STRUCTURE output was performed using CLUMPP and DISTRUCT [38–39]. CLUMPP permutes the clusters’ output by performing multiple replicate runs for the selected *K*. Samples with an average pairwise similarity (H value) of over 85% in one of the *K* clusters were considered to belong to that particular population; all other samples were considered admixed samples. DISTRUCT performs geographical displays of the aligned cluster assignment. We confirmed the optimal *K*, i.e. the *K* with the highest pseudo-F statistic, using AMOVA-based *K*-means clustering analysis in GENODIVE V2.0b23 (OS X 10.6 operating system). This method divides a number of individuals into an *a priori* assigned number of clusters (*K*) in such a way that minimizes within-group diversity and maximizes between-group diversity. The pseudo-F statistic was calculated by setting up simulated annealing runs with 150,000 steps and 50 algorithm repetitions to determine optimal clustering (highest *pseudo*- F-statistic).

eBURST v3 was used to identify clusters of closely related haplotypes, or haplogroups (HGs), which were defined as haplotypes sharing at least 9 loci from the 14 MS analyzed [40]. Haplotypes unrelated to any haplogroup (HG) were classified as singletons. The relationship between haplotypes following the defined *K* clusters was further analyzed by PHYLOViz [41]. Finally, to investigate the relationship between geographic and genetic distances in the study population, we performed principal coordinate analysis with the Mantel test for matrix correspondence in GenAlEx 6.5 [26]. Allele frequency was calculated in GenAlEx 6.5 using the predominant allele data set with 14 loci. The existence of a recent population bottleneck was investigated by evaluating the allele frequency distribution in the population (alleles at low frequencies are less abundant in populations with a recent bottleneck) [42–43].

## Results

### Baseline characteristics

In total, 234 individuals with *P*. *vivax* mono infection from the 260 recruited in the original study [12] were successfully genotyped and included in the analysis. Baseline characteristics of study participants are described in Table 1.

**Table 1:** Baseline characteristics of study patients.

Community	Sample	EM	Age groups		Sex		Hamlets	HH	Season of collection		AS	MP	MG
			≤15y	>15 y	M	F			Dry	Rainy			
Community 1 (%)	92 (39.3)	Cadong	55 (23.5)	37 (15.8)	51 (21.8)	41 (17.5)	4 (28.6)	45 (36.3)	16 (6.8)	76 (32.5)	59 (25.2)	7872	652
Community 2 (%)	57 (24.4)	Cadong	34 (14.5)	23 (9.8)	35 (15.0)	22 (9.4)	2 (14.2)	30 (24.2)	11 (4.7)	46 (19.7)	36 (15.4)	5829	1162
Community 3 (%)	32 (13.7)	Cadong	26 (11.1)	6 (2.6)	21 (8.9)	11 (4.7)	5 (35.8)	23 (18.5)	5 (2.1)	27 (11.5)	19 (8.1)	7144	490
Community 4 (%)	53 (22.6)	M'nong	29 (12.4)	24 (10.3)	33 (14.1)	20 (8.5)	3 (21.4)	26 (21.0)	6 (2.6)	47 (20.1)	24 (10.3)	5297	516
Total (%)	234 (100)		144 (61.5)	90 (38.5)	140 (59.8)	94 (40.2)	14 (100)	124 (100)	38 (16.2)	196 (83.8)	138 (59.0)	6691	723

EM, ethnic minority; HH, household; AS, asymptomatic infection (defined as no fever at enrolment), MP, mean number of asexual parasites per μL; MG, mean number of gametocytes per μL.

### Genetic diversity

Successful genotyping, with at least 12 of the 14 MS, was achieved in 194 patients (82.9%). Allele data were successfully recovered in more than 83% of the samples for all MS except MS20 and Pvsal1814, for which successful amplification was achieved in 66% and 75% of samples respectively.

The MS characteristics are described in Table 2. Overall, genetic diversity was moderate, with an average *He* = 0.68 (95%CI 0.58–0.77) for all MS. MS3 and MS9 were the least polymorphic markers (*He* = 0.45 and 0.31 respectively), while MS10 and Pvsal1814 were the most polymorphic markers (*He* = 0.99 and 0.92 respectively), which were therefore used to calculate MOI. The number of alleles per MS ranged from 3 to 14. All MS had non-significant differences in *He* values in the database containing all alleles per locus and the predominant allele datasets ruling out bias in the construction of haplotypes from polyclonal infections (*p* = 0.68). The average number of alleles per locus was 5.5 (95%CI 3.82–7.17), the average number of alleles detected in a sample by any locus was 1.14 (95%CI 1.0–1.27) and the average allelic richness was 5.05 (95%CI 4.11–5.98).

**Table 2:** Characteristics of the 14 microsatellite loci used in *P*. *vivax* populations from four communities in Quang Nam, Vietnam (n = 234).

Locus	Repeat sequence	ASR	No of alleles	H_E_	A*	PI/locus (%)	hMOI	Average alleles/ locus
MS1	(GAA)11	211–239	5	0.664	4.94	3.41	3	1.04
MS2	(TAAA)2TATA (TAAA)6 TATA (TAAA)19	179–215	4	0.686	4.00	5.12	2	1.06
MS3	(GAA)11	185–199	3	0.446	3.00	4.27	2	1.11
MS4	(AGT)18	189–237	6	0.728	5.94	20.94	3	1.24
MS5	CCTCTT(CCT)11	164–206	5	0.697	4.94	1.70	2	1.01
MS6	(TCC)2(TCT)3(CCT)2(TTC)2 GCTTCT(TCC)10	242–254	5	0.655	5.00	5.55	2	1.06
MS7	(GAA)9	142–157	3	0.601	3.00	5.55	2	1.06
MS8	(CAG)2(CAA)11	198–268	6	0.703	5.94	5.55	2	1.06
MS9	(GGA)18	155–170	3	0.311	3.00	2.56	2	1.03
MS10	GAA(GGA)2AGA(GGA)9AGA(GGA)4AGAGGAAGA(GGA)3AGAGGAAGA(GGAAAA)4(GGA)2(AGA)11(GGA)3(AGA)2GGAAGA(GGA)2	184–222	9	0.993	8.94	9.82	3	1.12
MS12	(TTC)10(TGC)4	207–224	5	0.618	5.00	5.55	2	1.06
MS15	(TCT)10	241–281	5	0.525	5.00	5.12	2	1.05
MS20	(GAA)11GAG(GAA)13(CAA)4GAA(CAA)5	148–206	4	0.680	4.00	3.41	2	1.05
Pvsal 1814	(AGA)44	546–704	14	0.919	7.99	53.84	3	2.01
All samples		142–704	77	0.676	5.05	71.37	2.2	1.14

ARS, Allele size range; A, Allelic richness; hMOI, highest multiplicity of infection; PI, Polyclonal infection.

* Predominant alleles only

The proportion of polyclonal infections, similar in all communities (*p* >0.05), was 71.4% (167/234) when all 14 MS were used, but 64.1% (141/220) (N = 220 as 14 samples had missing data for MS10 and Pvsal1814) when only Pvsal1814 and MS10, the most polymorphic markers were used. The same two MS were used to calculate MOI (based on 220 samples with completed data), whose mean in the four communities was 1.91 (95% CI 1.81–2.02), with no significant differences between communities (*p* = 0.52), age groups (MOI_≤15years_ = 2.0 vs MOI_>15years_ = 1.9, *p* = 0.50), gametocyte carriage (MOI_gametocytes present_ = 1.93 vs MOI_gametocytes absent_ = 1.80, *p* = 0.42), sex (MOI_male_ = 1.89 vs MOI_female_ = 1.95, *p* = 0.55), symptomatic vs asymptomatic (defined as fever at enrolment vs no fever at enrolment, MOI = 1.98 vs MOI = 1.80, respectively) (*p* = 0.10), season (MOI_rainy season_ = 1.90 vs MOI_dry seaoson_ = 1.97, *p* = 0.64), and ethnic minority (MOI_Cadong_ = 1.89 vs MOI_M’nong_ = 2.0, *p* = 0.46). No significant differences were found between the four communities for either level of *He* (*p* = 0.08) or allelic richness (*p* = 0.31).

We identified 101 haplotypes from 144 samples of which 84 haplotypes were defined as unique haplotype with complete genotyping data for 13 MS. MS20 was excluded because it had the lowest successful genotyping rate (66%). Of these haplotypes, 25.7% (26/101) were found in monoclonal infections and 16.8% (17/101) were found in both monoclonal and polyclonal infections; 6.93% of haplotypes (7/101) had a frequency of over 2 in 40 samples and the two most frequent haplotypes were detected in 7.6% (11/144) and 6.2% (9/144) of samples. One haplotype was shared between the four communities, 4 haplotype found in community 1 were also present in community 2 and one haplotype shared between community 3 and community 4.

**Fig 2:**
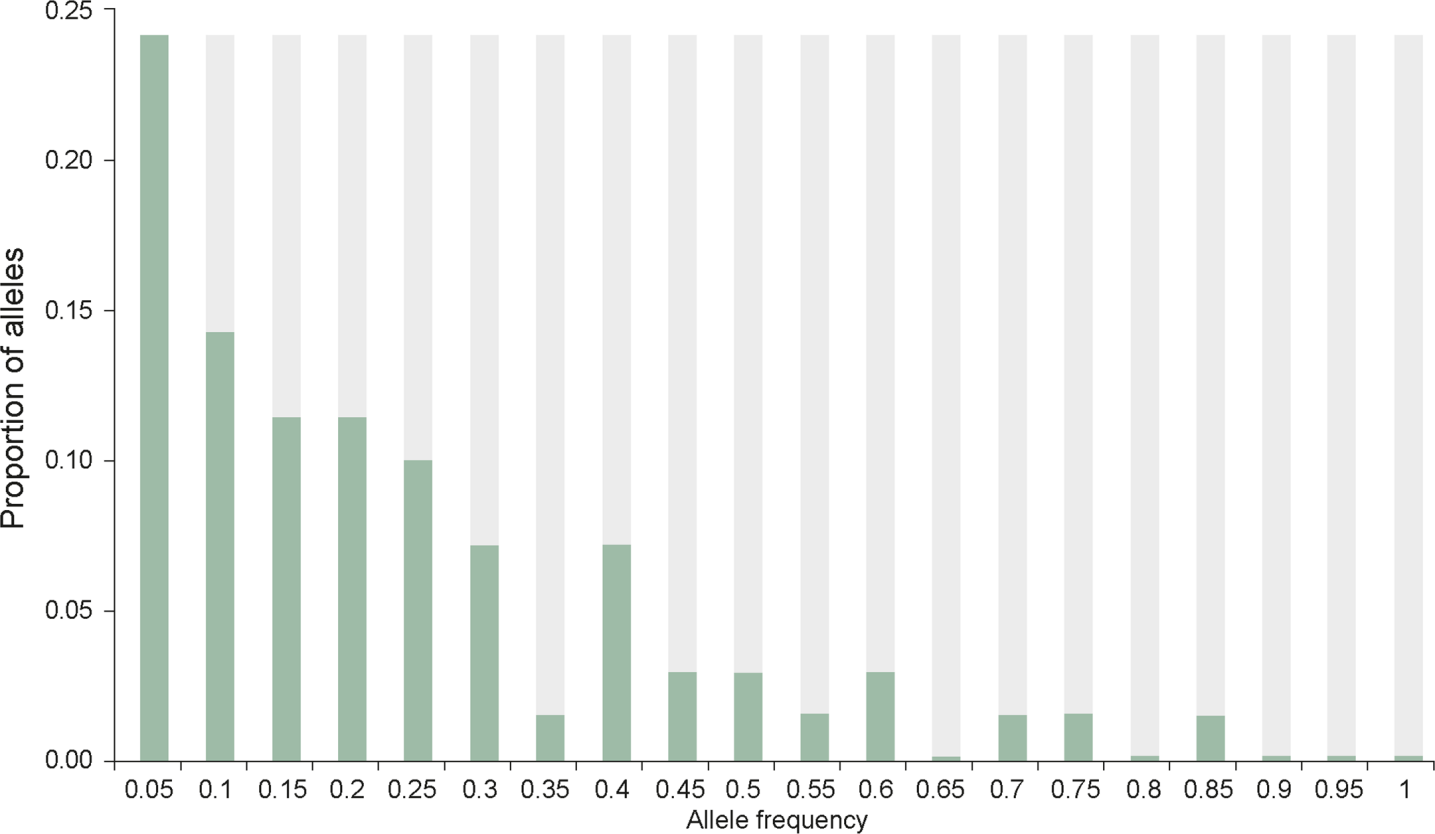
Allele frequency distribution in four communities in Quang Nam (n = 234). Predominant allele database and all loci combined are included.

To evaluate the existence of a recent population bottleneck we analyzed the allele frequency distribution in the population. Fig 2 shows an L-shaped distribution of allele frequencies, as would be expected from neutral evolution.

### Linkage disequilibrium

**Fig 3:**
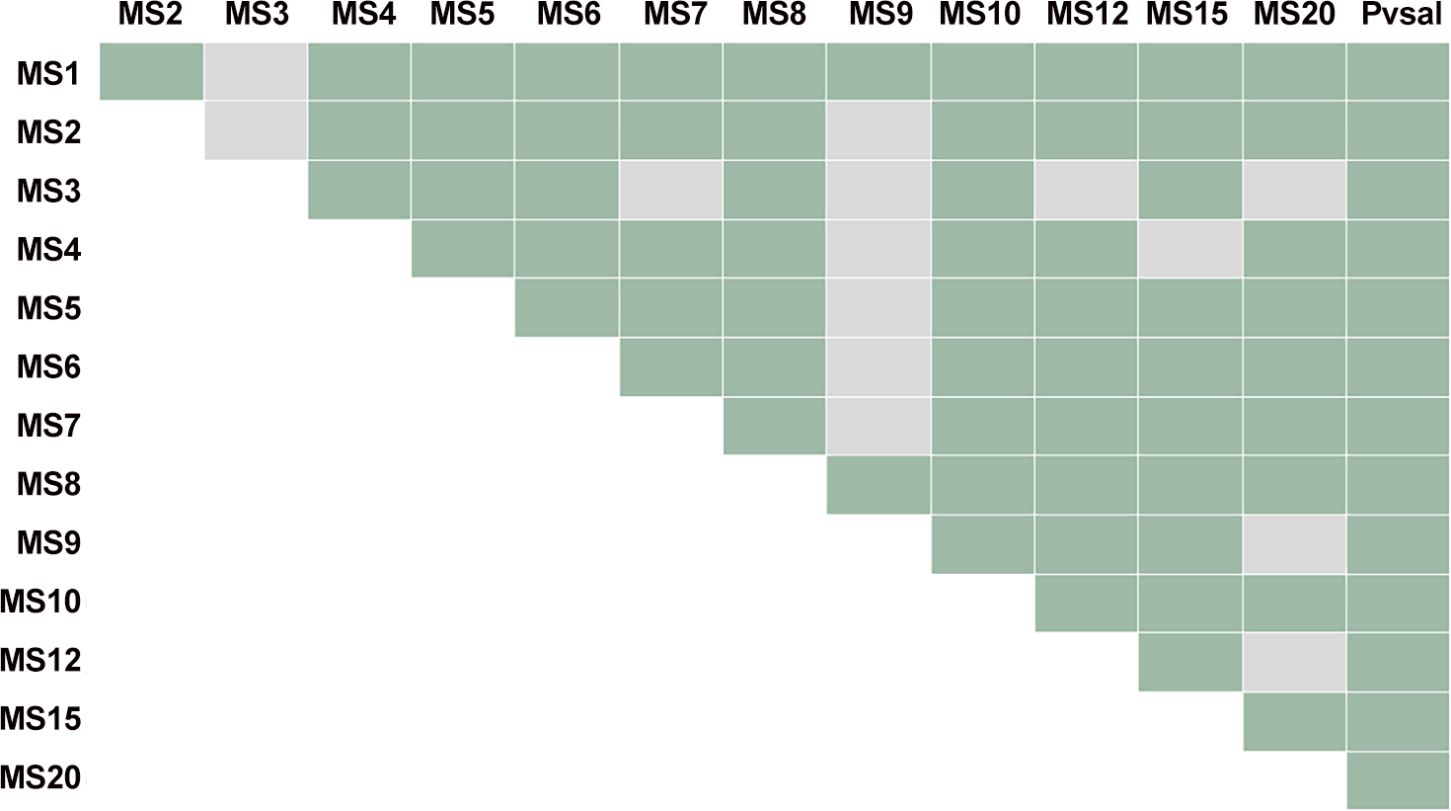
Pair-wise loci linkage disequilibrium analysis (N = 234). Significant association between alleles at pairs of loci in each sample was tested using FSTAT. Green color denotes LD at 5% based on 36400 permutations. Markers within the same contig are: MS4-MS5, MS7-MS8, MS12-MS15.

MS20 was also excluded from the LD analysis to maximize sample size and avoid bias due to an imbalanced number of samples between communities. Hence at least 25% of samples per community were included in the analysis. Significant LD was observed in each community ($I_{A}^{s}$ ranged from 0.10 to 0.17) and in the overall study population ($I_{A}^{s}$ = 0.113, *p* < 0.001). LD remained significant ($I_{A}^{s}$ = 0.059, *p* < 0.001) when only the unique haplotypes were used. We then examined patterns of LD between pairs of MS (Fig 3). Pairwise LD was observed between loci located within the same contigs (MS4-MS5, MS7-MS8, and MS12-MS15) and also within different contigs. Even though lower pairwise LD was observed in communities 3 and 4, the fact that the overall LD was significant (p = 0.008) suggests the existence of a clonal parasite population.

### Genetic differentiation

We first compared the datasets containing only monoclonal infections (n = 67) with the predominant allele (n = 234) and found low genetic differentiation (*F*_*ST*_ = 0.05), indicating absence of bias. Then, we calculated *F*_*ST*_ values for pairwise genetic differentiation between the four communities (Table 3). We observed moderate genetic differentiation between community 4 and the other communities (*F*_*ST*_ = 0.15–0.18) and low differentiation for the other combinations (*F*_*ST*_ < 0.1) (n = 144), indicating that the parasite population in community 4 is moderately, although significantly, differentiated from parasite populations in communities 1, 2, and 3 (*p* = 0.008). Similar *F*_*ST*_ values were obtained when only unique haplotypes (n = 84) were used.

**Table 3:** Pairwise genetic differentiation.

Standardize *F*_*ST*_	Community 1	Community 2	Community 3	Community 4
Community 1		0.046*	-0.028	0.153*
Community 2	0.043		-0.005	0.176*
Community 3	0.026	-0.035		0.168*
Community 4	0.174*	0.080*	0.145*	

The upper-right section shows the standardized fixation index (*F*_ST_) values obtained from 13 microsatellite markers (n = 144, MS20 excluded). The lower-left section shows *F*_ST_ values obtained from unique haplotypes from 13 microsatellite markers (n = 84).

* P value <0.05

### Population structure

**Fig 4:**
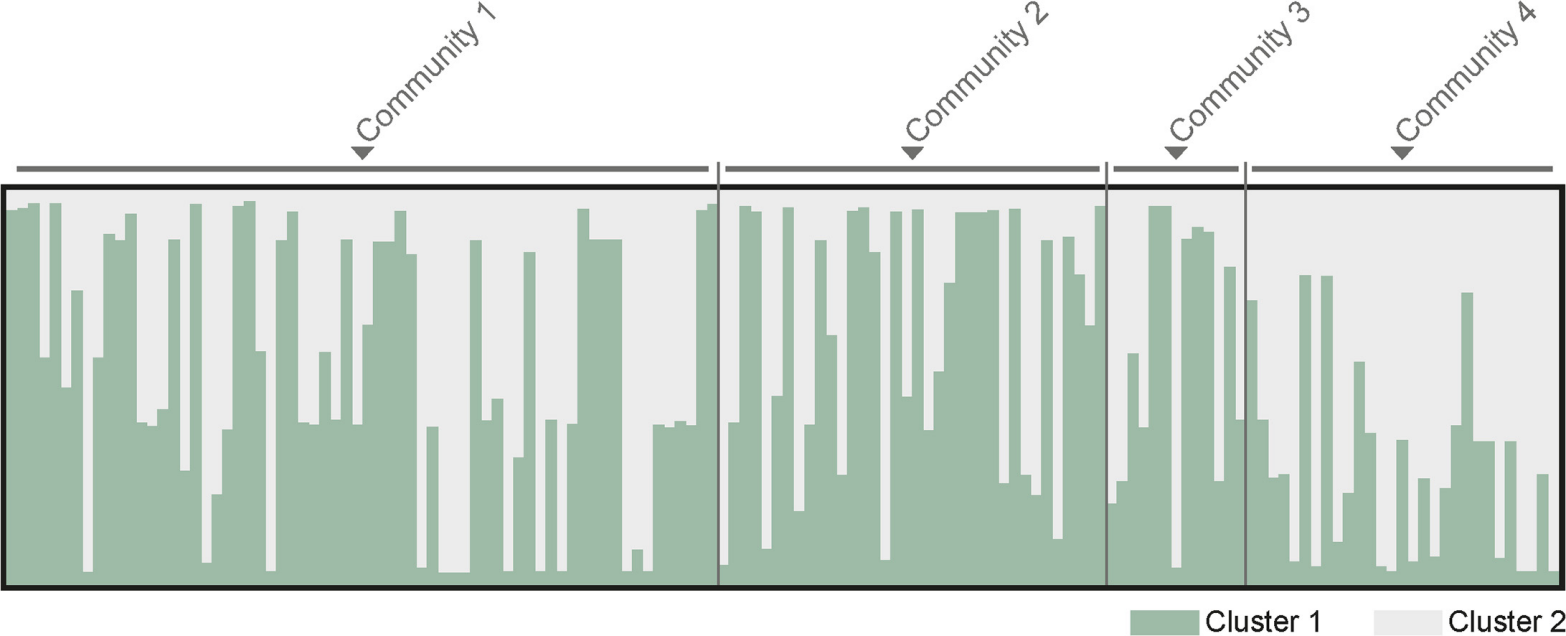
Genetic clustering analysis by STRUCTURE. The graph represent the clustering model when the parasite population was grouped into 2 clusters (*K* = 2). In the bar plot, each isolate is represented by a single vertical line divided into *K* colors.

Structure analysis identified the most likely clusters in the population to be (i) *K* = 7 (ΔK = 9.4), (ii) *K* = 2 (ΔK = 5.2), and (iii) K = 3 (ΔK = 3.2) (n = 144). The AMOVA-based *K*-means clustering analysis identified *K* = 2 as the optimal number of clusters (pseudo-*F* = 30.5). We further analyzed the parasite population divided by *K* = 2 (cluster 1 and cluster 2) with CLUMPP and DISTRUCT (Fig 4), and found that 33.3% (48/144) of the samples (with complete haplotypes) observed in the study population belonged to cluster 1 and 20.1% (29/144) belonged to cluster 2 and 46.6% (67/144) were admixed samples. Community 4 had the highest proportion of admixed samples (62.1%), followed by community 3 (53.9%), while community 1 and 2 had similar rates (40.9% and 41.3% respectively). The proportion of admixed samples remained high when the number of clusters was set to K = 3 and K = 7 (48.6% and 38.9%, respectively). Of note, cluster 1 samples were absent from community 4, which supports a moderate degree of population structure between communities 1–3 and community 4. However, principal coordinate analysis failed to detect geographical clustering (per community) in the population.

**Fig 5:**
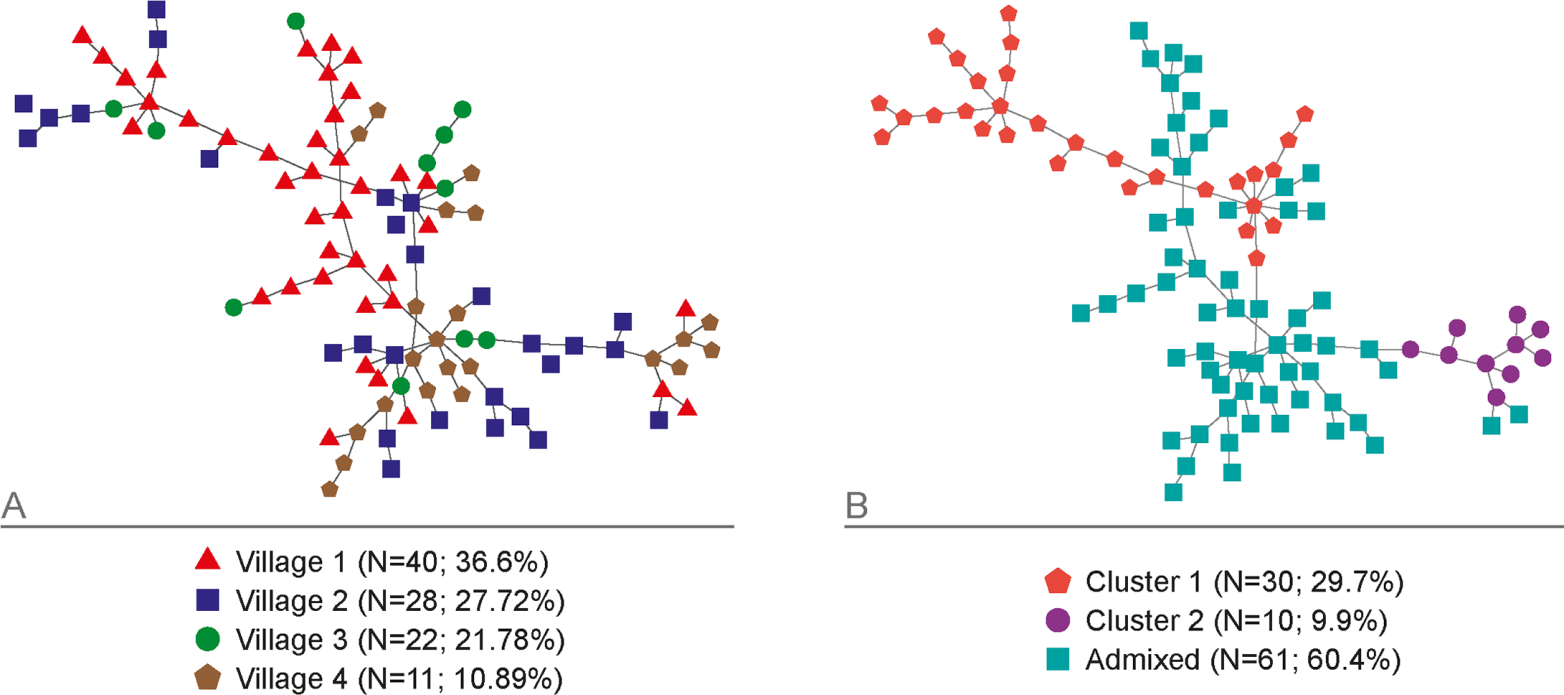
Phylogenetic relationship of 101 unique *P*. *vivax* haplotypes using PHYLOViZ. Haplotypes relationships by (A) community, and (B) clustering *K* = 2. Each colored figure represents a unique haplotype and grey lines indicate shared alleles among individual haplotypes.

Then, we investigated genetic relatedness, defined as haplotypes sharing at least 10/13 loci (n = 101) by eBURST. Eight different HGs and 13 singletons were identified. HG1 contained 51.5% (52/101) of all haplotypes in the four communities, while the other 7 HGs contained between 2.0% (2/101) to 11.9% (12/101) haplotypes. However, when relatedness was defined as haplotypes sharing at least 9/13 loci, only 2 HGs and 2 singletons were identified. HG1 included 95.0% (96/101) of all haplotypes detected. PHYLOVIZ analysis supported the existence of related haplotypes among all study communities (with slightly clustering of community 4 samples) (Fig 5A) and confirmed the absence of cluster 1 haplotypes in community 4 (Fig 5B).

## Discussion

We analyzed the genetic diversity and population structure of 234 *P*.*vivax* pre-treatment clinical isolates collected in a forested area of Central Vietnam between April 2009 and December 2010 [12]. We observed moderate levels of heterozygosity in all four study communities, with a high proportion of polyclonal infections and significant LD, suggestive of inbreeding across parasite populations circulating in the study communities. Genetic differentiation and population structure between study communities was low but present between villages at each side of the river defining a moderate geographical barrier to gene flow.

In this study we used eight MS (MS1, MS4, MS6, MS9, MS10, MS12, MS15, and MS20) with balanced diversity, three (MS2, MS5 and Pvsal1814) with unbalanced diversity, one (MS8) with significant excess diversity, and two (MS3 and MS7) with significant reduced diversity [25]. Mean *He* in the study population (*He* = 0.68) using those 14 MS was non-significantly different to *He* when only MS with balanced diversity (recommended for measuring population diversity) were used. Therefore, all MS were kept in the analysis to assess both diversity parameters and polyclonal infections, but only the two most polymorphic markers (MS10 and Pvsal1814) were kept to investigate MOI.

The mean *He* in our study population (*He* = 0.68) was similar to figures seen in areas of north-west Brazil with similar transmission intensities (*He* = 0.74 and *He* = 0.68) [43–44]; higher than those observed in South Korea (*He* = 0.43) [45] and the Loreto district, Peru (*He* = 0.37) [16], and lower than those seen in Sri Lanka (*He* = 0.89) [46], Pursat, Cambodia (*He* = 0.84) [16], and Binh Thuan, Central Vietnam (*He* = 0.88) [13]. In our study MS9, MS3, and MS7 displayed the lowest number of alleles per locus (n = 3) and MS9 and MS3 had the lowest *He* values (*He*_MS9_ = 0.31 and *He*_MS3_ = 0.45). MS3, MS7, and MS9 would therefore appear to be poorly informative markers in the study area and their use in future studies is not recommended.

Polyclonal infections were frequent (71.4%) when the results from 14 MS were combined and moderately lower (64.1%) when just Pvsal1814 and MS10 were used [47]. Mean MOI was 1.91, with similar MOI observed in symptomatic and asymptomatic study participants, possibly because of the high parasite density (mean 3,919/μL; 95%CI 2,852–4,986) detected in asymptomatic participants at day 0. In the literature, the proportion of polyclonal infections vary considerably depending on the MS markers used [16, 48], highlighting the need for a standardized methodology that allows comparison between studies and geographical regions. High proportions of polyclonal infections have also been reported in hypo-endemic areas in Sri Lanka (60%) [49], Colombia (60–80%) [48], the Amazon Basin in Brazil (50%) [43], and more recently, in a pre-elimination context in Sri Lanka (69%) [46]. It is noteworthy in a study carried out (1999–2000) in Binh Thuan province, central-south Vietnam, where the entomological inoculation rate was estimated at 1 infective bite/person/year, 100% of vivax infections were polyclonal with a mean MOI of 3.7 [13]. The high levels of genetic diversity and polyclonal infections in low transmission areas [13,23,48] can be, at least partially, explained by the unique biology of *P*.*vivax* which result in (i) a high prevalence of asymptomatic and low parasite density infections (which last longer because are difficult to detect, increasing the likelihood of repeated infections with divergent clones, resulting in increased polyclonality) and (ii) relapse from dormant liver stages (the reactivation of heterologous clones increases the likelihood of peripheral superinfections). Since a high proportion of study participants were asymptomatic at recruitment (59.0%) and poor adherence to PQ radical cure is known in the study area [3], the high proportion of polyclonal infections found in this study may reflect peripheral superinfection fed by heterologous clones from both relapses and reinfections.

Despite those high rates of polyclonal infections, we observed a significant LD ($I_{A}^{s}$ = 0.113, *p* < 0.001) in the overall study population. Asexual clones present in one infection produce gametocytes that, taken by the vector, recombine during meiosis and generate new haplotypes in a process known as outcrossing. Consequently, the breakdown of pre-existing associations between unlinked loci would reduce LD to low levels [50] as opposed to recombination between gametes from the same parasite [51]. As transmission decreases, fewer parasite types will be present in the population and recombination will often occur between related parasites, increasing the level of inbreeding in the population. This is supported by the fact that 53.8% of all polyclonal infections were identified by multiple alleles at just one locus. Indeed, LD remained significant in the analysis using only unique haplotypes, indicating that it is a result of inbreeding rather than expansion of few haplotypes due to outbreaks or epidemics [31]. Closely related parasites in hypoendemic areas have been previously reported [52, 53]. In addition, inbreeding was further supported by overall significant pairwise LD [21,31]. LD combined with high levels of polyclonality has been reported in rural Amazonia [54] and more recently in Sri Lanka [46]. The authors of these studies offered two alternative interpretations for this phenomenon. First, the MS may not be strictly neutral (10/14 MS mapping to loci encoding either hypothetical or annotated proteins may be subject to natural selection) [22–23]. And second, replication-slippage events during mitotic (asexual) replication could result in the generation of new alleles due to the addition or deletion of repeats [49,55]. If the replication-slippage rate is higher than that of effective recombination (the probability of producing a recombinant genome), the clones generated would increase polyclonality, without altering LD.

It has been previously reported that replication-slippage events (and therefore number of alleles per locus and *He*) correlate positively with increasing repeat length and non-perfect repeats motifs, i.e. interrupted or compound motifs [25,56]. Pvsal1814 MS used in this study, which had an (AGA)_44_ motif structure with an interrupted/compound motif, *He* = 0.91 and 14 different alleles with frequencies ranging from 1.3% to 16%, identified 53.8% of all polyclonal samples in the study population. Indeed, inherent mutability in this MS has been described to produce excess diversity, which in turn is recommended to identify MOI [25].

We identified 101 haplotypes, of which 84 appeared only once in the population. Ninety percent of them were grouped in a single haplogroup (HG1), defined by identical alleles in at least 9/13 loci, indicating a high degree of relatedness among parasites across the communities. These results support the view that despite a high level of polyclonality, inbreeding among highly related haplotypes maintains LD.

The adjusted genetic differentiation was low between communities 1, 2, and 3 (*F*_*ST*_ < 0.05) and moderate when community 4 was included (*F*_*ST*_ *=* 0.15–0.18), indicating limited geographical boundaries between neighboring communities 1–3 but higher differentiation with the community across the river. In concordance with the *F*_*ST*_ values, the STRUCTURE analysis detected two main parasite populations. Two clusters of haplotypes, with a high proportion of mixture haplotypes (60.4%) were observed in all four communities. The fact that a majority of haplotypes found in community 4 belonged to cluster 2, which was the minor cluster in the other 3 communities, supports a certain degree of differentiation between communities 1–3 and 4. Moderate population differentiation between these communities can be explained by geographical proximity and socioeconomic relationships between the communities’ inhabitants as previously described [3]. Inhabitants of community 1–3 (located at one side of the river) belong to the Cadong ethnic group and therefore share some degree of kinship, facilitating social exchange. Conversely, community 4, whose inhabitants belong to the M’nong ethnicity, is located at the other side of the river with limited access during the rainy season.

Malaria incidence in the Quang Nam province has dropped by 78.0% over the last decade thanks to the implementation of efficient control strategies [1,17,57]. At the time of the study, malaria prevalence in the study area was 7.8% as assessed by light microscopy and 23.6% as estimated by PCR [3]. Therefore, the moderate-to-high levels of genetic diversity detected, together with the high polyclonality and low population structure are consistent with an epidemiological context of transition from moderate to low endemicity [58–59].

Future studies aiming at identifying changes in genetic diversity and population structure to support the development or improvement of control and elimination interventions should include isolates collected at several time points from all areas where malaria is prevalent (or has been recently eliminated). Ideally, a molecular surveillance system should be implemented within the existing network of sentinel sites for drug resistance across the country to support evaluation of interventions and improve response strategies at the provincial level.

Parasite populations with strong LD and the presence of gene flow could fuel the spread of resistant parasites in the event of the emergence of drug resistance, threatening current treatment efforts and achievements towards malaria elimination in Central Vietnam. Temporal analysis to investigate haplotype persistence and the risk of clonal expansion is urgently needed in order to inform decision makers.
